# miRNAexpression profile of retinal pigment epithelial cells under oxidative stress conditions

**DOI:** 10.1002/2211-5463.12360

**Published:** 2018-01-02

**Authors:** Luigi Donato, Placido Bramanti, Concetta Scimone, Carmela Rinaldi, Rosalia D'Angelo, Antonina Sidoti

**Affiliations:** ^1^ Department of Biomedical and Dental Sciences and Morphofunctional Imaging Division of Medical Biotechnologies and Preventive Medicine University of Messina Italy; ^2^ Department of Cutting‐Edge Medicine and Therapies Biomolecular Strategies and Neuroscience Section of Neuroscience‐applied, Molecular Genetics and Predictive Medicine I.E.ME.S.T. Palermo Italy; ^3^ IRCCS Centro Neurolesi ‘Bonino‐Pulejo’ Messina Italy

**Keywords:** miRNA, regulation, retina, retinal degeneration, RNA‐Seq

## Abstract

Deep analysis of regulative mechanisms of transcription and translation in eukaryotes could improve knowledge of many genetic pathologies such as retinitis pigmentosa (RP). New layers of complexity have recently emerged with the discovery that ‘junk’ DNA is transcribed and, among these, miRNAs have assumed a preponderant role. We compared changes in the expression of miRNAs obtained from whole transcriptome analyses, between two groups of retinal pigment epithelium (RPE) cells, one untreated and the other exposed to the oxidant agent oxidized low‐density lipoprotein (oxLDL), examining four time points (1, 2, 4 and 6 h). We found that 23 miRNAs exhibited altered expression in the treated samples, targeting genes involved in several biochemical pathways, many of them associated to RP for the first time, such as those mediated by insulin receptor signaling and son of sevenless. Moreover, five RP causative genes (*KLHL7*,* RDH11*,*CERKL*,* AIPL1* and *USH1G*) emerged as already validated targets of five altered miRNAs (hsa‐miR‐1307, hsa‐miR‐3064, hsa‐miR‐4709, hsa‐miR‐3615 and hsa‐miR‐637), suggesting a tight connection between induced oxidative stress and RP development and progression. This miRNA expression analysis of oxidative stress‐induced RPE cells has discovered new regulative functions of miRNAs in RP that should lead to the discovery of new ways to regulate the etiopathogenesis of RP.

**Abbreviations**
AIPL1: aryl hydrocarbon receptor interacting protein like 1
CERKL: ceramide kinase like
EDGE: empirical analysis of DGE
ER: endoplasmic reticulum
IRS: insulin receptor substrate
KLHL7: kelch‐like protein 7
miRNome: miRNA transcriptome
oxLDL: oxidized low‐density lipoprotein
RDH11: retinol dehydrogenase 11
RPE: retinal pigment epithelium
RP: retinitis pigmentosa
SOS: son of sevenless
USH1G: usher syndrome type 1G

Analysis of regulative mechanisms of transcription and translation in eukaryotes is a very challenging task. New layers of complexity are daily discovered, such as the preponderant role in regulative functions of miRNAs. miRNAs represent a group of short non‐coding RNAs that induce transcript degradation or translational inhibition of their target mRNAs, acting as post‐transcriptional regulators of gene expression 1. They assume the role of key regulators of several important biological processes, in both physiological and pathological conditions 2, controlling specific pathways by targeting networks of functionally correlated genes. Alterations of miRNA expression, due to mutations in either the miRNA itself or its target genes, could lead to several pathological conditions such as cancers 3, neurodegenerative and genetic diseases 4. Therefore miRNAs, due to their emerging role as disease biomarkers, might be possible therapeutic targets in human disorders 5.

The retina is the back portion of the eye, photosensitive and able to focus light signals towards the optic nerve first, then towards the brain, after transduction of them into electrical stimuli. This light‐sensitive layer of the eye represents the target of a huge number of human inherited pathologies, such as retinitis pigmentosa (RP) 6. RP is a genetic disease that determines retinal degeneration by inducing a slow and progressive death in photoreceptors and retinal pigment epithelium (RPE) cells 7, leading to loss of ability to transmit to the brain the visual information. The term ‘pigmentosa’ deals with the characteristic appearance, during the advanced states of the disease, of abnormal areas of pigment in the retina. Much evidence supports the role of miRNAs in normal retinal development and functions 8. Alterations of miRNA regulation in conditional Dicer mouse mutant eyes reduce and damage normal development of lens, cornea, retina and optic chiasm 9. Furthermore, post‐developmental disruption of miRNA processing in photoreceptors leads to severe functional impairments 10. Interestingly, the targeted deletion of specific retina‐enriched miRNAs has relevant effects on vertebrate eye 11, such as pathogenic roles in human retinal diseases 12. Nowadays, miRNA expression data come only from the analysis of murine miRNA transcriptome (miRNome) but, due to structural and functional differences between human and mouse retinas, they are not totally useful. Therefore, an improved knowledge of human retina miRNome, especially of patients affected by retinal disease, could lead to a better understanding of the physiopathology of this tissue. In this work, we investigated the complexity of human retina miRNome, analyzing data from human RPE cell transcriptomes. RPE represents a single layer of post‐mitotic cells, acting as both a selective barrier to and a vegetative regulator of the overlying photoreceptor layer, thereby playing a key role in its maintenance. Due to its specific proteins, RPE helps to renew outer segments by phagocytizing the spent discs of photoreceptor outer segments, regulates the trafficking of nutrients and waste products to and from the retina, protects the outer retina from excessive high‐energy light and light‐generated reactive oxygen species and maintains retinal homeostasis through the release of diffusible factors. In detail, we compared miRNA expression changes between two group of RPE cells, one exposed to the oxidant agent oxidized low‐density lipoprotein (oxLDL) and the other untreated, considering four time points (1, 2, 4 and 6 h) over basal one (time zero). oxLDL was chosen because it has already been tested on several neurodegenerative diseases but, above all, because it was seen that high cholesterol level could be linked to RP development and progression 13. The main purpose of our work was to discover which miRNAs changed during oxidative stress induction and what their targets are, in order to better understand how reactive oxygen species might lead to RP development.

## Materials and methods

This study was approved by the Ethics Committee of Azienda Policlinico Universitario ‘G. Martino’ Messina.

### Cell culture

Human RPE‐derived cells (H‐RPE – human retinal pigment epithelial cells, Clonetics™, Lonza) were maintained at 1 × 10^6^ cells·mL^−1^ in culture in T‐75 flasks containing RtEGM™ retinal pigment epithelial cell growth medium BulletKit® (Clonetics™, Lonza, Basel, Switzerland) with 2% fetal bovine serum, 100 units·mL^−1^ of penicillin and 100 μg·mL^−1^ of streptomycin and incubated at 37 °C with 5% CO_2_. After 24 h, 100 μg·mL^−1^ of oxLDL was added to the treated group.

### Total RNA sequencing

RNA was extracted by TRIzol™ reagent (Invitrogen, Thermo Fisher Scientific, Waltham, MA, USA), following manufacturer's protocol, and quantified with a Qubit 2.0 fluorimeter by Qubit® RNA assay kit (Thermo Fisher Scientific). Expression analysis was realized comparing human RPE cells treated with 100 μg·mL^−1^ of oxLDL and untreated ones, both at the moment of treatment and for four different time points (1, 2, 4 and 6 h). Libraries were generated using 1 μg of total RNA and the Ion Total RNA‐Seq Kit v2 (Thermo Fisher Scientific), then purified by Dynabeads® magnetic beads and quantified with a Qubit 2.0 fluorimeter with Qubit dsDNA HS Assay Kit. An appropriate library amount was used for clonal amplification performed with the Ion PI™ Template OT2 200 Kit v2 (Thermo Fisher Scientific) on the Ion One Touch™ 2 System; template‐positive Ion Sphere Particles were enriched with the Ion One Touch™ Enrichment System. Sequencing runs were performed on an Ion Proton™ Sequencer (Ion Torrent technology; Thermo Fisher Scientific), using the Ion PI™ Sequencing 200 Kit v2 and the Ion PI™ Chip Kit v2 (Thermo Fisher Scientific). The experiment was repeated three times.

### Quality validation and read mapping

Sequence reads were generated from RPE‐specific cDNA libraries on the Ion Torrent Proton. Obtained raw sequences were filtered to remove low quality reads (average per base Phred score < 28). Furthermore, the reads containing adaptor sequences and low‐quality sequences (reads presenting ambiguous bases denoted as ‘N’) were also trimmed from the raw data. The quality of analyzed data was checked using fastqc (v.0.11.5) and qualimap (v.2.2.1) software. The filtered data were then analyzed by clc genomics workbench v.10.0.1 (Qiagen Aarhus, Denmark; https://www.qiagenbioinformatics.com/products/clc-genomics-workbench/) using *Homo sapiens* genome hg19 and Ensembl RNA database v.74 as references. RNA‐Seq analysis was conducted using the following settings: quality trim limit = 0.01, ambiguity trim maximum value = 2. Map to annotated reference: minimum length fraction and minimum similarity fraction = 0.8, maximum number of hits/read = 2, type of organism = eukaryote, paired settings =default.

### Small RNA analysis

The applied approach counted the different types of small RNAs in the data and compared them with databases of miRNAs or other small RNAs. Once whole RNA‐Seq data were imported, the small RNAs were extracted and counted, in order to create a small RNA sample that could be used for further steps. Sequences were filtered based on length (reads below 15 bp and above 55 bp were discarded) and on minimum sampling count (set at 1). The aligned and selected reads were grouped on the sequence of the mature miRNAs allowing up to two mismatches within the exact length of the reference mature sequence (i.e. excluding trimming or extension variants). Subsequently, the number of reads mapping on each mature miRNA was counted and then normalized using either the trimmed mean of M‐values (TMM) method 14 or reads per million (CPM). Finally, the small RNA sample produced when counting the tags was enriched by comparing the tag sequences with the annotation resources miRBase (v21) and Ensembl non‐coding RNA database (v74).

### Gene expression and statistical analysis

The original expression values were log2 transformed and normalized in order to ensure that samples are comparable and assumptions on the data for analysis are met 15. In order to highlight the miRNAs with different level of expression between untreated and treated samples, and for the four considered time points, we categorized them into two groups, based on count ratios (fold‐change): (a) up‐regulated (fold change > 1); (b) down‐regulated (0 < fold change < 1). Furthermore, because the fold changes are linear, for any value smaller than 1 (i.e. for down‐regulation), we chose to replace the value by its negative reciprocal value, in order to make the variation more noticeable (for instance, 2‐fold downregulation is indicated by a value of ‐2 instead of 0.5). Due to the few biological replicates available for each of the experimental groups studied (only three replicates for each considered time point), but with many features to be studied simultaneously (miRNAs in a whole transcriptome), we applied the empirical analysis of DGE (EDGE) statistical algorithm, which implements the ‘exact test’ for two‐group comparisons developed by Robinson and Smyth 16. The test is based on the assumption that the count data follow a negative binomial distribution, which in contrast to the Poisson distribution has the characteristic that it allows for a non‐constant mean–variance relationship. The ‘exact test’ of Robinson and Smyth is similar to Fisher's exact test, but also accounts for over‐dispersion caused by biological variability. The miRNAs uniquely identified in the RPE cells with at least five unique gene reads, greater than one‐fold (up‐regulated) or lower than one‐fold (down‐regulated) changes in expression based on the ratio of expression values, and with Bonferroni‐adjusted *P* values lower than 0.05 were selected for functional categorization of differentially expressed miRNAs.

### Validation of miRNAs by qRT‐PCR

To confirm the transcriptome results, the 23 analyzed miRNA were validated by qRT‐PCR. Complementary DNA synthesis from miRNA samples was performed using the miScript II RT Kit and HiSpec Buffer (Qiagen). The obtained cDNA was subjected to the RT‐PCR in the ABI 7500 fast sequence detection system (Applied Biosystems, Thermo Fisher Scientific), using the BRYT‐Green based PCR reaction. PCR amplification was performed in a total reaction mixture of 20 μL, containing 20 ng cDNA, 10 μL 2× GoTaq1qPCR Master Mix (Promega, Madison, WI, USA) and 0.2 μm of each primer. The PCR was run with the standard thermal cycle conditions using the two‐step qRT‐PCR method: an initial denaturation at 95 °C for 30 s, followed by 40 cycles of 30 s at 95 °C and 30 s at 60 °C. Each reaction was run in triplicate, considering all selected time points (1, 2, 4 and 6 h), and the average threshold cycle (*C*_t_) was calculated for each replicate. The expression of miRNAs was calculated relative to the expression level of the endogenous control U6, and the relative expression of a gene was calculated using the $2^{-\Delta \Delta C_{t}}$ method. The correlation of the fold‐change of the gene expression ratios between qRT‐PCR and RNA‐Seq was checked by linear regression analysis in spss Statistics v24.0 software (IBM Corp., Armonk, NY, USA).

### miRNA target identification and pathway analysis

Obtained miRNAs were firstly analyzed by the experimentally validated miRNA‐target interactions database miRTarBase (http://mirtarbase.mbc.nctu.edu.tw) 17, and then investigated by microT v5.0 (http://diana.imis.athena-innovation.gr) 18 web‐based prediction software, in order to discover new potential targets. Once targets were obtained, a pathway analysis of selected miRNAs was performed by diana‐mirpath v3.0 (http://snf-515788.vm.okeanos.grnet.gr) 19 and cluego cytoscape plugin 20.

## Results

### Sequencing analysis and mapping statistics

RNA sequencing carried out with Ion Torrent yielded a total of 11 214 300 quality reads (mean mapping quality = 32.92) with mean read length of 155.03 bp. All reads were previously aligned to GRCh37/hg19 reference assembly, and then to known human miRNAs (miRBase v21) 21 and GRCh37 non‐coding RNAs 22. About 71 500 small RNAs were founded in all samples, 69 158 of which were annotated and about 2341 unannotated. In details, 23 miRNAs, each one considered as a ‘group’ made of known precursors and/or mature miRNAs resulting from mapping (Table 1), showed expression alterations at the analyzed time points (Table S1). All previous mapping statistics are based on average values calculated for all three replicates in each time point. Detailed information on RNA‐Seq statistics are available in Table 2.

**Table 1:** Altered miRNAs with ranking. The RNA‐Seq analysis highlighted the 23 grouped miRNAs (mature, precursors and precursor variants) with expression alterations, ranked on their abundance (based on read count). As seen in table, the 10 top‐ranked miRNAs accounted for almost 80% of the total count, and the top five for 60%

Rank	ID	Sequence	Length (bp)	Count (precursors)	Count (precursors with variants)	Count (mature)	Total
1	hsa‐mir‐3615	UCUCUCGGCUCCUCGCGGCUC	21	7	13	9	29
2	hsa‐mir‐3654	GACUGGACAAGCUGAGGAA	19	26	6	7	39
3	hsa‐mir‐198	GGUCCAGAGGGGAGAUAGGUUC	22	8	4	5	17
4	hsa‐mir‐5047	UUGCAGCUGCGGUUGUAAGGU	21	7	4	5	16
5	hsa‐mir‐671‐3p	UCCGGUUCUCAGGGCUCCACC	21	9	4	5	18
6	hsa‐mir‐1307‐3p	ACUCGGCGUGGCGUCGGUCGUG	22	0	7	3	10
7	hsa‐mir‐1181	CCGUCGCCGCCACCCGAGCCG	21	3	1	2	6
8	hsa‐mir‐3655	GCUUGUCGCUGCGGUGUUGCU	21	0	3	2	5
9	hsa‐mir‐4315	CCGCUUUCUGAGCUGGAC	18	2	1	2	5
10	hsa‐mir‐7705	AAUAGCUCAGAAUGUCAGUUCUG	23	1	1	2	4
11	hsa‐mir‐3064‐3p	UUGCCACACUGCAACACCUUACA	23	1	0	1	2
12	hsa‐mir‐3198	GUGGAGUCCUGGGGAAUGGAGA	22	2	0	1	3
13	hsa‐mir‐3917	GCUCGGACUGAGCAGGUGGG	20	1	0	1	2
14	hsa‐mir‐4523	GACCGAGAGGGCCUCGGCUGU	21	1	0	1	2
15	hsa‐mir‐4647	GAAGAUGGUGCUGUGCUGAGGAA	23	1	0	1	2
16	hsa‐mir‐4709‐3p	UUGAAGAGGAGGUGCUCUGUAGC	23	0	2	1	3
17	hsa‐mir‐4721	UGAGGGCUCCAGGUGACGGUGG	22	0	2	1	3
18	hsa‐mir‐4800‐3p	CAUCCGUCCGUCUGUCCAC	19	1	1	1	3
19	hsa‐mir‐6084	UUCCGCCAGUCGGUGGCCGG	20	1	0	1	2
20	hsa‐mir‐637	ACUGGGGGCUUUCGGGCUCUGCGU	24	1	0	1	2
21	hsa‐mir‐6501‐3p	CCAGAGCAGCCUGCGGUAACAGU	23	2	0	1	3
22	hsa‐mir‐8085	UGGGAGAGAGGACUGUGAGGC	21	0	1	1	2
23	hsa‐mir‐922	GCAGCAGAGAAUAGGACUACGUC	23	0	1	1	2
Total				74	51	55	180

**Table 2:** RNA‐Seq statistics. Considering the whole experiment, about 6000 small RNAs were detected (average value), of which about 96% was annotated by Ensembl GRCh37 non‐coding RNA database and miRBase, with an average reads count of about 70 000. All average values were calculated considering the means of three replicates for each time point

Annotation	Small RNA count	Percentage	Read count	Percentage	Perfect matches	Percentage	1 mismatches	Percentage	2 mismatches	Percentage
Annotated	5624	96	69 158	96.7	2843	50.6	1549	27.5	1232	21.9
With miRBase (*Homo sapiens*)	9	0.2	115	0.2	5	55.6	2	22.2	2	22.2
With *Homo_sapiens* GRCh37.ncrna	5615	99.8	69 043	99.8	2834	50.6	1547	27.5	1230	21.9
Unannotated	235	4	2341	3.3						
Total	5859	100	71 499	100						

### Expression analysis

**Figure 1:**
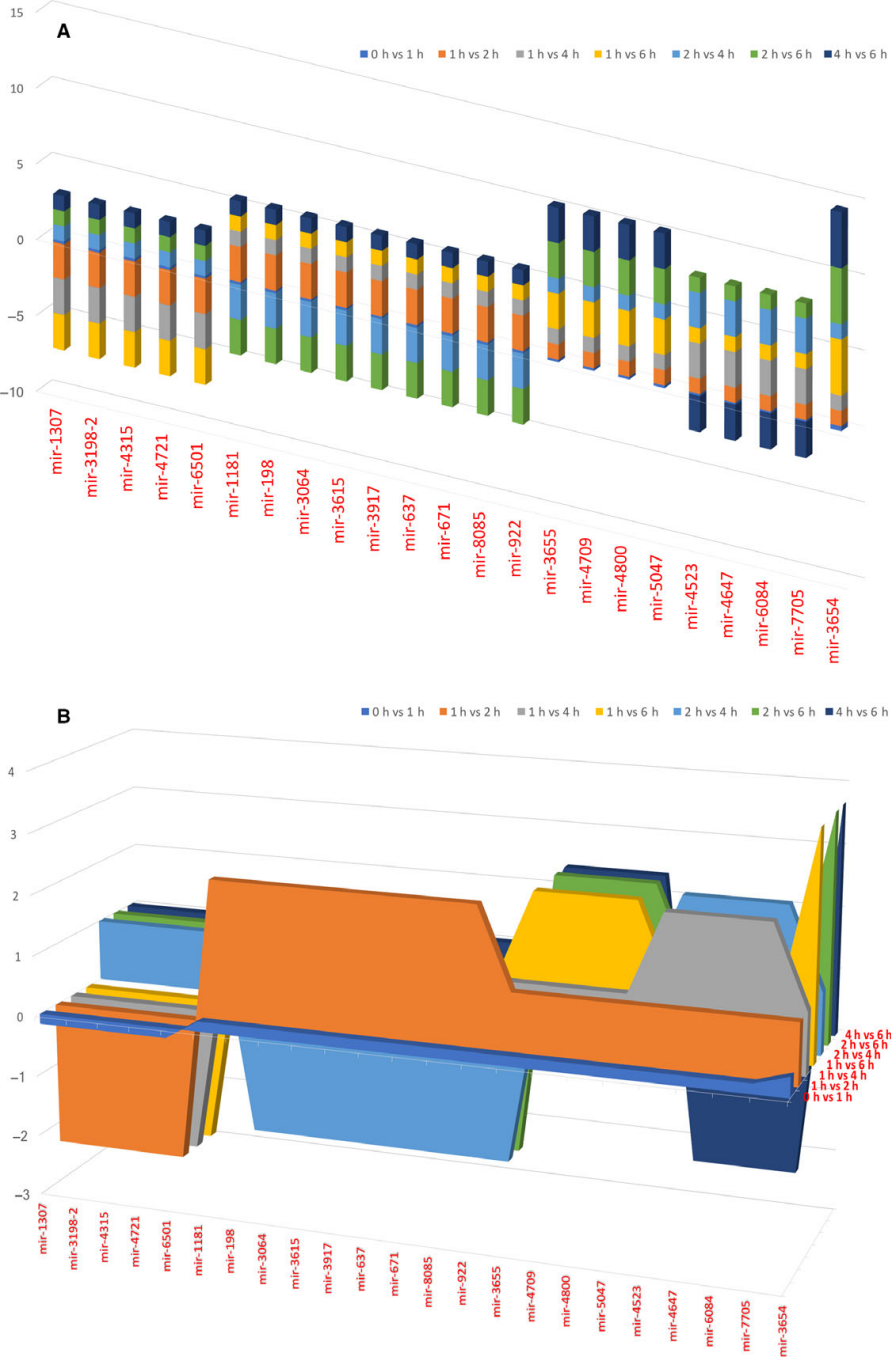
Bar graph and 3D area graph of miRNA fold‐change trends during analyzed time points. 3D bar graph (A) and 3D area graph (B) of selected miRNA fold‐change throughout all considered time points. Five clusters emerged from the analysis: the first two exhibited an opposite trend, globally negative for cluster 1 (mir‐1307, mir‐3198‐2, mir‐4315, mir‐4721, mir‐6501) and globally positive for cluster 2 (mir‐1181, mir‐198, mir‐3064, mir‐3615, mir‐3917, mir‐637, mir‐671, mir‐8085, mir‐922); the last two clusters evidenced a strong positive trend, greater in cluster 5 (mir‐3655, mir‐4709, mir‐4800, mir‐5047) than in cluster 4 (mir‐4523, mir‐4647, mir‐6084, mir‐7705). The only cluster with its own individual trend was the third, made of the single mir‐3654, with a globally slightly positive trend, which did not show noticeable changes due to the opposite expression differences in the last two time points. For any value smaller than 1, we chose to replace the value by its negative reciprocal value, in order to make the variation more noticeable. More details on single miRNA expressions are presented in Table 3.

The predominant length of the resulting mature miRNAs was 21 bp. We identified a total of 55 mature miRNAs with an average expression level of three reads across all considered treated and untreated RPE cells cultures (Table 1). The variability was low across samples, with an expected higher trend for miRNAs expressed at lower levels. As highlighted in Table 3 and in Fig. 1, there are few values of fold‐change that repeat during analyzed time points, with the highest value of 3.7 reached by mir‐3654. Interestingly, we found five clusters of miRNAs showing particular trends through analyzed time points. Cluster 1 (mir‐1307, mir‐3198‐2, mir‐4315, mir‐4721 and mir‐6501) showed a small decrease (fold change = −0.2) in treated samples after 1 h, followed by a greater diminution (fold change = −2.3) from 2 h up to 6 h. Cluster 2, showing the highest number of deregulated miRNAs (mir‐1181, mir‐198, mir‐3064, mir‐3615, mir‐3917, mir‐637, mir‐671, mir‐8085 and mir‐922), evidenced a small increase (fold change = +0.2) in treated samples after 1 h, then increased (fold change = +2.3) up to 2 h, and finally diminished to a similar extent (fold change = −2.3) at 4 h, remaining unchanged up to 6 h. Cluster 4 (mir‐4523, mir‐4647, mir‐6084 and mir‐7705) showed a small increase (fold change = +0.2) in treated samples after 1 h, remained constant up to 2 h, then increased noticeably (fold change = +2.3) at 4 h, and decreased again (fold change = −2.3) at 6 h. Cluster 5 (mir‐3655, mir‐4709, mir‐4800 and mir‐5047) showed a small increase (fold change = +0.2) in treated samples after 1 h, remained constant up to 4 h, then increased noticeably (fold change = +2.3) at 6 h. Cluster 3, made of the single mir‐3654, presented a small increase (fold change = +0.3) in treated samples after 1 h, remained constant up to 4 h, then increased noticeably (fold change = +3.7) at 6 h (Fig. 1). We ranked the expressed miRNAs based on their abundance (Table 1). The 10 top‐ranked miRNAs accounted for almost 80% of the total count and the top five for 60%.

**Table 3:** miRNA expression variations throughout all analyzed time points. All 23 selected miRNAs showed particular fold‐change trends, between treated and untreated samples, during considered time points (0, 1, 2, 4 and 6 h), with a few values that are repeats. The whole results were statistically validated by Bonferroni‐corrected EDGE test, and *P* values are reported

Sequence	Length	Name	0 h vs 1 h	EDGE test (*P* value), Bonferroni corrected	1 h vs 2 h	EDGE test (*P* value), Bonferroni corrected	2 h vs 4 h	EDGE test (*P* value), Bonferroni corrected	4 h vs 6 h	EDGE test (*P* value), Bonferroni corrected	1 h vs 4 h	EDGE test (*P* value), Bonferroni corrected	1 h vs 6 h	EDGE test (*P* value), Bonferroni corrected	2 h vs 6 h	EDGE test (*P* value), Bonferroni corrected
CCGUCGCCGCCACCCGAGCCG	21	hsa‐mir‐1181	0.166666667	3.65711 × 10^−5^	2.332680923	3.65711 × 10^−5^	−2.332680923	3.65711 × 10^−5^	1	4.74338 × 10^−20^	1	0	1	4.74338 × 10^−20^	−2.332680923	3.65711 × 10^−5^
ACUCGGCGUGGCGUCGGUCGUG	22	hsa‐mir‐1307‐3p	−0.166666667	3.65711 × 10^−5^	−2.332680923	3.65711 × 10^−5^	1	0	1	4.74338 × 10^−20^	−2.332680923	3.65711 × 10^−5^	−2.332680923	3.65711 × 10^−5^	1	4.74338 × 10^−20^
GGUCCAGAGGGGAGAUAGGUUC	22	hsa‐mir‐198	0.166666667	3.65711 × 10^−5^	2.332680923	3.65711 × 10^−5^	−2.332680923	3.65711 × 10^−5^	1	3.65711 × 10^−5^	1	0	1	3.65711 × 10^−5^	−2.332680923	4.74338 × 10^−20^
UUGCCACACUGCAACACCUUACA	23	hsa‐mir‐3064‐3p	0.166666667	3.65711 × 10^−5^	2.332680923	3.65711 × 10^−5^	−2.332680923	3.65711 × 10^−5^	1	4.74338 × 10^−20^	1	0	1	4.74338 × 10^−20^	−2.332680923	3.65711 × 10^−5^
GUGGAGUCCUGGGGAAUGGAGA	22	hsa‐mir‐3198	−0.166666667	3.65711 × 10^−5^	−2.332680923	3.65711 × 10^−5^	1	0	1	4.74338 × 1^0−20^	−2.332680923	3.65711 × 10^−5^	−2.332680923	3.65711 × 10^−5^	1	4.74338 × 10^−20^
UCUCUCGGCUCCUCGCGGCUC	21	hsa‐mir‐3615	0.166666667	3.65711 × 10^−5^	2.332680923	3.65711 × 10^−5^	−2.332680923	0	1	4.74338 × 10^−20^	1	3.65711 × 10^−5^	1	3.65711 × 10^−5^	−2.332680923	4.74338 × 10^−20^
GACUGGACAAGCUGAGGAA	19	hsa‐mir‐3654	0.333333333	0.000182855	1	0.000182855	1	7.31422 × 10^−5^	3.694855996	0.000109713	1	0.000109713	3.694855996	4.74338 × 10^−20^	3.694855996	0.000182855
GCUUGUCGCUGCGGUGUUGCU	21	hsa‐mir‐3655	0.166666667	3.65711 × 10^−5^	1	3.65721 × 10^−5^	1	0	2.334587154	4.74338 × 10^−20^	1	3.65711 × 10^−5^	2.334587154	3.65711 × 10^−5^	2.334587154	4.74338 × 10^−20^
GCUCGGACUGAGCAGGUGGG	20	hsa‐mir‐3917	0.166666667	3.65711 × 10^−5^	2.332680923	3.65711 × 10^−5^	−2.332680923	3.65711 × 10^−5^	1	4.74338 × 10^−20^	1	0	1	4.74338 × 10^−20^	−2.332680923	3.65711 × 10^−5^
CCGCUUUCUGAGCUGGAC	18	hsa‐mir‐4315	−0.166666667	3.65711 × 10^−5^	−2.332680923	3.65711 × 10^−5^	1	0	1	4.74338 × 10^−20^	−2.332680923	3.65711 × 10^−5^	−2.332680923	3.65711 × 10^−5^	1	4.74338 × 10^−20^
GACCGAGAGGGCCUCGGCUGU	21	hsa‐mir‐4523	0.166666667	0	1	0	2.332680923	3.65711 × 10^−5^	−2.332680923	3.65711 × 10^−5^	2.332680923	3.65711 × 10^−5^	1	4.74338 × 10^−20^	1	4.74338 × 10^−20^
GAAGAUGGUGCUGUGCUGAGGAA	23	hsa‐mir‐4647	0.166666667	0	1	0	2.332680923	3.65711 × 10^−5^	−2.332680923	3.65711 × 10^−5^	2.332680923	3.65711 × 10^−5^	1	4.74338 × 10^−20^	1	4.74338 × 10^−20^
UUGAAGAGGAGGUGCUCUGUAGC	23	hsa‐mir‐4709‐3p	0.166666667	0	1	0	1	3.65711 × 10^−5^	2.335287929	3.65711 × 10^−^5	1	3.65711 × 10^−5^	2.335287929	4.74338 × 10^−20^	2.335287929	4.74338 × 10^−20^
UGAGGGCUCCAGGUGACGGUGG	22	hsa‐mir‐4721	−0.166666667	3.65711 × 10^−5^	−2.332680923	3.65711 × 10^−5^	1	0	1	4.74338 × 10^−20^	−2.332680923	3.65711 × 10^−5^	−2.332680923	3.65711 × 10^−5^	1	4.74338 × 10^−20^
CAUCCGUCCGUCUGUCCAC	19	hsa‐mir‐4800‐3p	0.166666667	3.65711 × 10^−5^	1	3.65711 × 10^−5^	1	3.65711 × 10^−5^	2.338006877	4.74338 × 10^−20^	1	0	2.338006877	4.74338 × 10^−20^	2.338006877	3.65711 × 10^−5^
UUGCAGCUGCGGUUGUAAGGU	21	hsa‐mir‐5047	0.166666667	3.65711 × 10^−5^	1	3.65731 × 10^−5^	1	0	2.335287929	4.74338 × 10^−20^	1	3.65711 × 10^−5^	2.335287929	3.65711 × 10^−5^	2.335287929	4.74338 × 10^−20^
UUCCGCCAGUCGGUGGCCGG	20	hsa‐mir‐6084	0.166666667	0	1	0	2.332680923	3.65711 × 10^−5^	−2.332680923	3.65711 × 10^−5^	2.332680923	3.65711 × 10^−5^	1	4.74338 × 10^−20^	1	4.74338 × 10^−20^
ACUGGGGGCUUUCGGGCUCUGCGU	24	hsa‐mir‐637	0.166666667	3.65711 × 10^−5^	2.332680923	3.65711 × 10^−5^	−2.332680923	3.65711 × 10^−5^	1	4.74338 × 10^−20^	1	0	1	4.74338 × 10^−20^	−2.332680923	3.65711 × 10^−5^
CCAGAGCAGCCUGCGGUAACAGU	23	hsa‐mir‐6501‐3p	−0.166666667	3.65711 × 10^−5^	−2.332680923	3.65711 × 10^−5^	1	0	1	4.74338 × 10^−20^	−2.332680923	3.65711 × 10^−5^	−2.332680923	3.65711 × 10^−5^	1	4.74338 × 10^−20^
UCCGGUUCUCAGGGCUCCACC	21	hsa‐mir‐671‐3p	0.166666667	3.65711 × 10^−5^	2.332680923	3.65711 × 10^−5^	−2.332680923	0	1	4.74338 × 10^−20^	1	3.65711 × 10^−5^	1	3.65711 × 10^−5^	−2.332680923	4.74338 × 10^−20^
AAUAGCUCAGAAUGUCAGUUCUG	23	hsa‐mir‐7705	0.166666667	0	1	0	2.332680923	3.65711 × 10^−5^	−2.332680923	3.65711 × 10^−5^	2.332680923	3.65711 × 10^−5^	1	4.74338 × 10^−20^	1	4.74338 × 10^−20^
UGGGAGAGAGGACUGUGAGGC	21	hsa‐mir‐8085	0.166666667	3.65711 × 10^−5^	2.332680923	3.65711 × 10^−5^	−2.332680923	3.65711 × 10^−5^	1	4.74338 × 10^−20^	1	0	1	4.74338 × 10^−20^	−2.332680923	3.65711 × 10^−5^
GCAGCAGAGAAUAGGACUACGUC	23	hsa‐mir‐922	0.166666667	3.65711 × 10^−5^	2.332680923	3.65711 × 10^−5^	−2.332680923	3.65711 × 10^−5^	1	4.74338 × 10^−20^	1	0	1	4.74338 × 10^−20^	−2.332680923	3.65711 × 10^−5^

### qRT‐PCR verification

**Figure 2:**
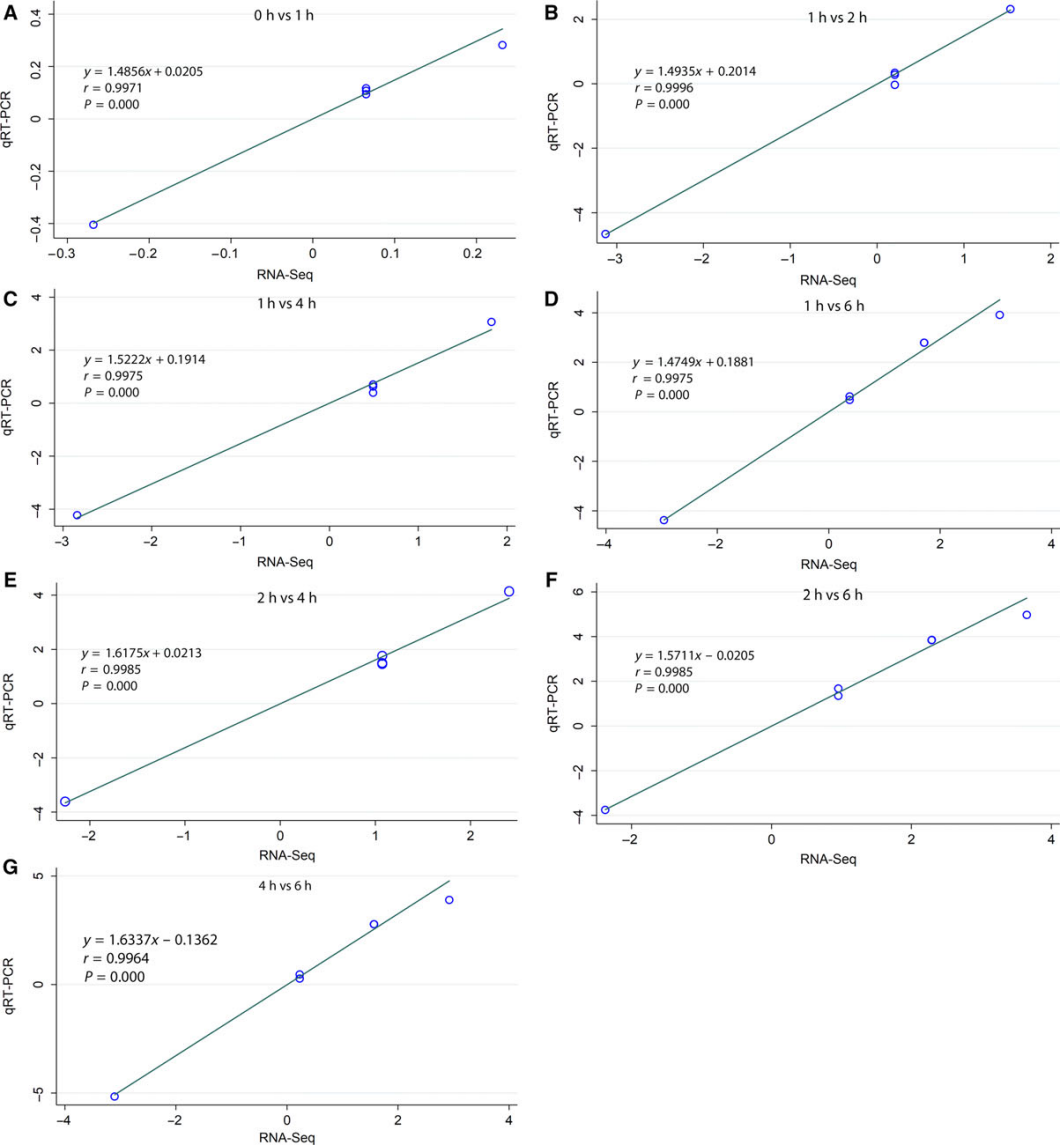
Correlation analysis of fold‐change data between qRT‐PCR and RNA‐Seq. Data from qRT‐PCR and RNA‐Seq are means of three replicates, considering all selected time points (A, B, C, D, E, F, G). Scatterplots were generated by the fold‐change values from RNA‐Seq (*x*‐axis) and qRT‐PCR (*y*‐axis).

To validate the authenticity and reproducibility of the RNA‐Seq results, the 23 analyzed miRNAs were selected for qRT‐PCR analysis, and the obtained expression profiles were similar to the results of transcriptome analysis (data not shown). The linear regression analysis showed a significantly positive correlation of the relationship between gene expression ratios of qRT‐PCR and RNA‐Seq for all selected time points (Fig. 2), confirming our transcriptomic data validity.

### miRNA target identification and pathway analysis

**Figure 3:**
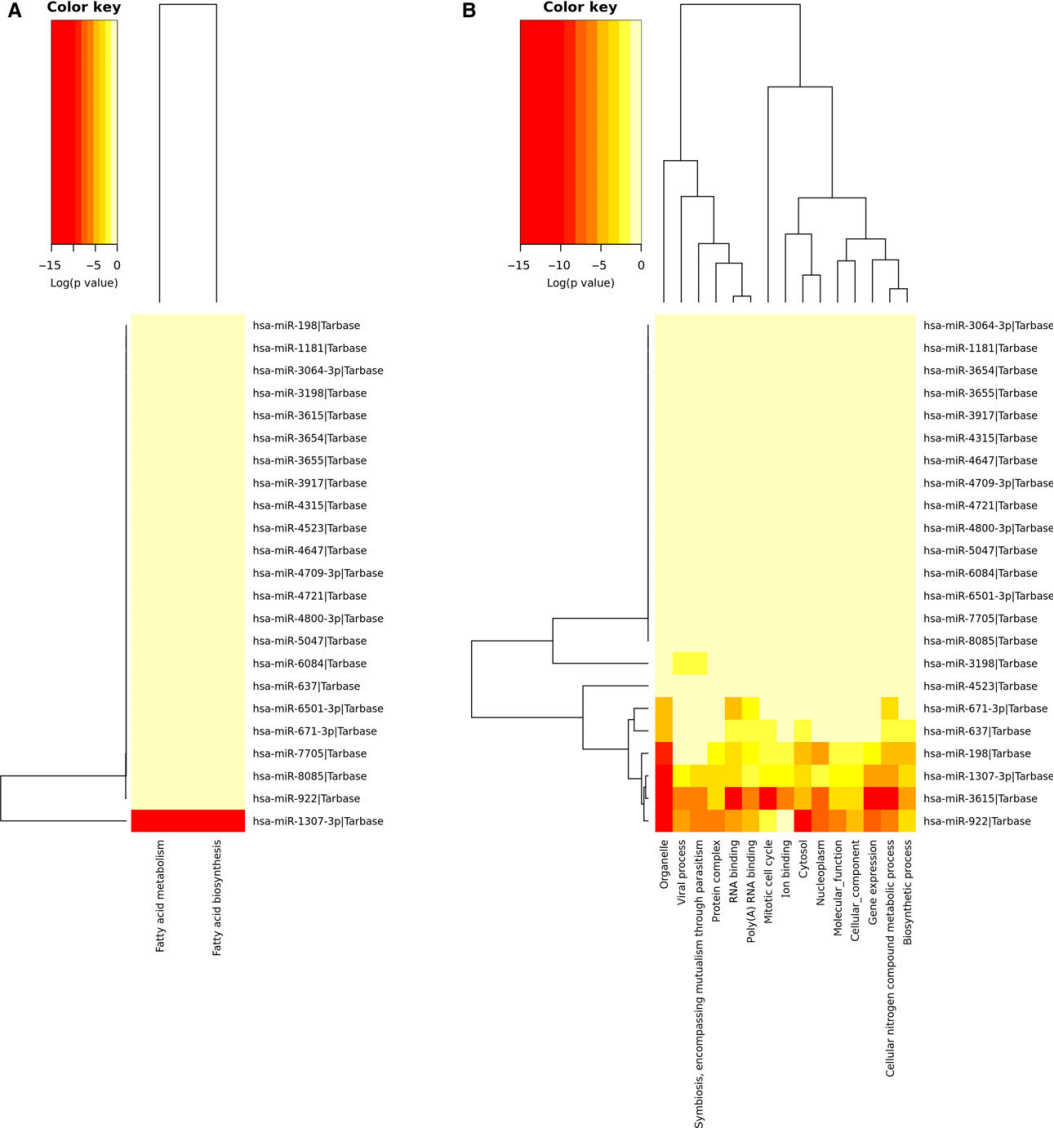
Hierarchical clustering of features by mirPath analysis. As evidenced in heat maps from KEGG (A) and GO (B) mirPath analysis, several predicted pathways (two for KEGG, 15 for GO) were statistically associated to considered miRNAs.

**Figure 4:**
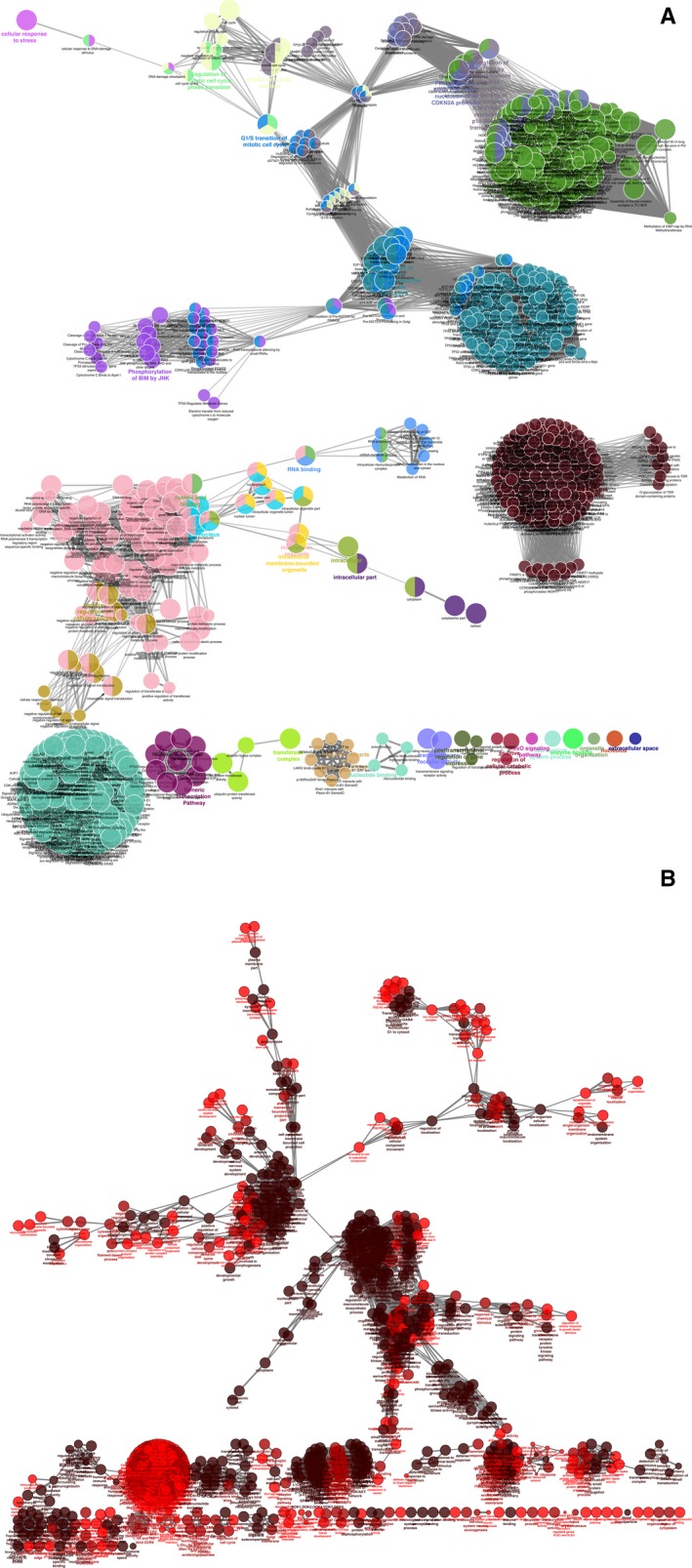
cluego pathway analysis of miRNAs target genes from miRTarBase and microT databases. cluego analysis highlighted a rich and very clustered network of possible involved pathways for both miRTarBase validated targets (A) and microT predicted ones (B). Details are given in Table S3.

There were 1402 genes that were experimentally validated targets of 23 miRNAs found altered in miR TarBase. Five miRNAs (hsa‐miR‐1307‐3p, hsa‐miR‐3064‐3p, hsa‐miR‐4709‐3p, hsa‐miR‐3615 and hsa‐miR‐637) are able to regulate five already known RP causative genes [Kelch‐like protein 7 (*KLHL7*), retinol dehydrogenase 11 (*RDH11*), ceramide kinase like (*CERKL*), aryl hydrocarbon receptor interacting protein like 1 (*AIPL1*) and usher syndrome type 1G (*USH1G*)], as demonstrated by high‐throughput sequencing of RNA isolated by cross‐linking immunoprecipitation; photoactivatable‐ribonucleoside‐enhanced cross‐linking and immunoprecipitation; and crosslinking, ligation and sequencing of hybrids. Eight hundred and ten genes, instead, were common to those that emerged from microT prediction, which in total showed 7351 target genes. Subsequently, mirPath Kyoto Encyclopedia of Genes and Genomes (KEGG) analysis showed a statistically significant association between six analyzed miRNAs (hsa‐miR‐3615, hsa‐miR‐922, hsa‐miR‐4523, hsa‐miR‐671‐3p, hsa‐miR‐198 and hsa‐miR‐1307‐3p) and ‘NF‐kappa B signaling pathway’, and two miRNAs (hsa‐miR‐922 and hsa‐miR‐1307‐3p) showed association with ‘fatty acids biosynthesis and metabolism’. mirPath gene ontology (GO) analysis, instead, showed a statistically significant association between six miRNAs (hsa‐miR‐3615, hsa‐miR‐922, hsa‐miR‐637, hsa‐miR‐671‐3p, hsa‐miR‐198 and hsa‐miR‐1307‐3p) and four categories (‘cellular nitrogen compound’, ‘metabolic process’, ‘organelle’, and ‘RNA binding and poly(A) RNA binding’), five miRNAs (hsa‐miR‐3615, hsa‐miR‐922, hsa‐miR‐637, hsa‐miR‐198 and hsa‐miR‐1307‐3p) and three categories (‘cytosol’, ‘biosynthetic process’ and ‘mitotic cell cycle’), and four miRNAs (hsa‐miR‐3615, hsa‐miR‐922, hsa‐miR‐198 and hsa‐miR‐1307‐3p) and six categories (‘gene expression’, ‘nucleoplasm’, ‘protein complex’, ‘molecular function’ and ‘cellular component’). Detailed results are available in Table S2 and Fig. 3. Once we had obtained experimental validated pathways, we enriched previously gathered data with pathway analysis based on miRTarBase and microT common gene targets, realized by cytoscape and its plugin cluego. Pathways with the highest number of involved miRNA‐regulated genes could be clustered in ‘intracellular organelle’, ‘regulation of cellular process’, ‘organic cyclic, hetero cycle, cellular nitrogen and aromatic compounds metabolic process’, ‘protein metabolic process’, ‘nucleic acid binding’, ‘macromolecule modification’, ‘regulation of transcription’, ‘regulation of response to stimulus’, ‘proteosomal cleavage of substrate’, ‘insulin receptor substrate (IRS) – and son of sevenless (SOS) ‐mediated signaling’, ‘endoplasmic reticulum (ER)–phagosome pathway ‘, ‘inflammation’, ‘ABC‐transporters disorders’, and ‘signaling by Hedgehog’. Detailed pathways, including the exact number of involved genes, are shown in Fig. 4.

## Discussion

Retinitis pigmentosa is an ocular disease with very heterogeneous phenotypes with unusually complex molecular genetic causes 23. Such an intricate picture, primarily determined by locus and allelic heterogeneity 24, is worsened by the actual lack of knowledge on all possible causative genes and their function. Moreover, little is known about involvement of regulative non‐coding RNAs 25, which are already considered the most promising targets of experimental therapies 26. Using high‐throughput sequencing technologies, we analyzed the whole transcriptome of RPE cells treated with oxLDL during a follow‐up of four time points (1, 2, 4 and 6 h) after exposure, and compared the results with untreated cells. Due to the high coverage of our sequencing experiment, we were able to detect miRNAs and sequence variants that had a low level of expression. Furthermore, the parallel analysis of three replicates for each selected group for each time point, along with a specific statistical test, allowed us to obtain reliable data, overcoming possible bias‐related variability in miRNA expression levels and nucleotide sequences. Oxidative stress plays a critical role in the etiopathogenesis of RP 27, leading to pathobiological changes including outer blood–retina barrier breakdown and senescence of RPE cells 28. RPE cells are very susceptible to oxidative stress, due to high metabolic activity, including physiological phagocytosis and life‐long light illumination 29. Therefore, as high cholesterol could be linked to RP development and progression 30, oxLDL could represent the source of pathobiological changes of RPE cells 31, such as outer blood–retina barrier dysfunction 32, inhibition of processing of photoreceptor outer segments by RPE 33, expression of transforming growth factor‐β 2 34, synthesis alterations of extracellular matrix components 35, increasing RPE apoptosis 36 and senescence changes 31. Moreover, several miRNAs were already associated to retinal development and function in vertebrates, such as miR‐216a and miR‐23a 37, 38, although at very low levels. Following these data, we noticed that about 74% of miRNAs found showed an average read count of 2 or even 1. The meaning of this observation could be that miRNAs with relatively low expression levels can play a tissue‐specific physiological role, underlining the importance of a high‐coverage for the study of human tissues. Furthermore, the analysis of miRNA sequence diversity indicated that a wide range of variants (with the highest peak of 13 precursor variants for hsa‐mir‐3615) are expressed in RPE cells, bearing different and often composite sequence modifications, many of which probably happened after oxidative stress induction. In order to evaluate how oxLDL could regulate miRNA expression, we hypothesized a possible ‘dual’ mechanism, direct and not. It was seen, from whole transcriptome analysis data, that *DICER1* was globally up‐regulated after induced oxidative stress. In detail, it showed the following fold‐change trend: 1 h → 2.181, 2 h → 2.614, 4 h → 1 and 6 h → 2.799. Therefore, the oxLDL probably regulates miRNAs both directly (especially as shown by down‐regulated miRNAs) and indirectly by Dicer (particularly when it is up‐regulated and over‐expresses miRNAs). Interestingly, the five clusters of selected miRNAs showed particular pairings: the first two clusters exhibited an opposite trend, globally negative for cluster 1, and globally positive for cluster 2; the last two cluster evidenced a strong positive trend, greater in cluster 5 than in cluster 4; the only cluster with its own individual trend was the third, with a globally slightly positive trend, which did not noticeably change, due to the opposite expression differences in the last two time points. In detail, basing on mirPath, the only ubiquitous miRNA is hsa‐mir‐1307, whose contribution could determine a decrease in all resultant pathways. However, because it is the only down‐expressed miRNA throughout all the time points, its role might be considered less decisive than the others. Therefore, a possible dysregulation of the nuclear factor kappa‐light‐chain‐enhancer of activated B cells (NF‐κB) signaling pathway emerged, a pathway already associated to retinal degeneration 39, 40, along with fatty acid metabolism, known to be one of the most involved pathways in RP 41 and its particular forms, such as Stargardt syndrome 42. Moreover, several new interesting pathways could be altered by the analyzed miRNAs, as emerged from target gene predictions. Cellular nitrogen compound metabolic processes seems to be impaired, as already highlighted in aqueous humor and peripheral blood of RP patients 43, leading to nitrosative stress. Additionally, alterations of metabolic processes involving organic cyclic, cellular aromatic and heterocycle compounds, such as reaction intermediates of the retinoid cycle, could lead to retinal degeneration 44. Furthermore, three signaling pathways were predicted to be altered by selected miRNA dysregulation: insulin receptor signaling, possibly related to glucose sequestration by RPE in RP dominant subjects 45, SOS‐mediated signaling, already associated to ocular manifestations in Noonan syndrome 46, 47, and Hedgehog signaling, whose impairments could lead to ciliary trafficking defects 48. The described pathways along with ABC‐transporter disorders 49, inflammation 50, ER–phagosome pathways 51 and the ubiquitin proteasome pathway 52 represent all possible targets of miRNA regulation in RPE cells. Finally, miRTarBase showed experimentally verified gene targets of several considered miRNAs, already known to be causative of different forms of RP: *KLHL7* (target of hsa‐miR‐1307), whose mutations could determine alterations in ubiquitination of target proteins for proteasome‐mediated degradation 53; *RDH11* (target ofhsa‐miR‐3064), encoding a fundamental enzyme needed for vision‐related and systemic retinoic acid metabolism 54; *CERKL* (target of hsa‐miR‐4709), encoding an antioxidant protein that is crucial to photoreceptor survival 55, 56; *AIPL1* (target of hsa‐miR‐3615), whose mutations cause various form of recessive RP and Leber congenital amaurosis 57; and *USH1G* (target of hsa‐miR‐637), one of best known causative gene of Usher syndrome type I 58.

## Conclusions

We analyzed whole transcriptomes of two group of RPE cells, treated with oxLDL and untreated, comparing miRNA expression changes at four selected time points (1, 2, 4 and 6 h) from time zero. We found that 23 grouped miRNAs exhibited expression alterations in treated samples, targeting genes involved in several biochemical pathways. Most of these, such as fatty acid metabolism and the ubiquitin proteasome pathway, are already known to be directly involved in retinal degenerations. Several others, instead, might be associated for the first time to RP etiopathogenesis, such as IRS‐ and SOS‐mediated pathways. Moreover, five RP causative genes (*KLHL7*,* RDH11*,* CERKL*,* AIPL1* and *USH1G*) emerged as already validated targets of five altered miRNAs (hsa‐miR‐1307, hsa‐miR‐3064, hsa‐miR‐4709, hsa‐miR‐3615 and hsa‐miR‐637), suggesting a tight connection between induced oxidative stress and RP development and progression, thanks to the important junction ring represented by regulative functions of miRNAs. Nevertheless, many other important aspects have to be investigated, such as variant miRNA targets and isomiR, along with predicted target validation. Additionally, we have to underline that predicted miRNA targets resulted from *in silico* analyses and, even if they are based on statistical significance algorithms and literature data, they will need to be experimentally validated. Furthermore, a deeper transcriptome sequencing on a larger number of samples could permit us to increase the number of detected miRNAs, improving knowledge of regulative functions of these small RNAs and RP.

## Author contributions

LD planned experiments and wrote the manuscript; PB contributed reagents; CS performed experiments; CR performed manuscript supervision; RD analyzed data; AS wrote and drafted the manuscript.

## Supporting information


**Table S1.** miRNAs precursors and precursors variants coming from RNA‐Seq analysis.

**Table S2.** mirPath KEGG and GO analysis.

**Table S3.** ClueGO detailed pathway analysis of miRNAs target genes from miRTarBase and microT databases.

**Table S1.** miRNAs precursors and precursors variants coming from RNA‐Seq analysis.
