# Corrigendum to: miRNA expression profile of retinal pigment epithelial cells under oxidative stress conditions

**DOI:** 10.1002/2211-5463.12526

**Published:** 2018-10-05

**Authors:** Luigi Donato, Placido Bramanti, Concetta Scimone, Carmela Rinaldi, Francesco Giorgianni, Sarka Beranova‐Giorgianni, Diwa Koirala, Rosalia D'Angelo, Antonina Sidoti

**Affiliations:** ^1^ Division of Medical Biotechnologies and Preventive Medicine Department of Biomedical and Dental Sciences and Morphofunctional Imaging University of Messina Italy; ^2^ Department of Cutting‐Edge Medicine and Therapies, Biomolecular Strategies and Neuroscience Section of Neuroscience‐Applied, Molecular Genetics and Predictive Medicine I.E.ME.S.T. Palermo Italy; ^3^ IRCCS Centro Neurolesi ‘Bonino‐Pulejo’ Messina Italy; ^4^ Department of Pharmaceutical Sciences University of Tennessee Health Science Center Memphis TN USA

The list of authors of the paper by Donato *et al*. 1 was incomplete. The full list of authors and their contributions are given here.

## Author contributions

LD planned experiments and wrote the manuscript. PB contributed reagents. CS performed experiments. CR performed manuscript supervision. FG conceived and designed the study, directed the cell culture experiments and obtained the RNA‐seq data. SBG designed the study. DK carried out cell culture and treatment, and RNA isolation. RD analysed data. AS wrote and drafted the manuscript.
