# Supplementary material for: miRNAexpression profile of retinal pigment epithelial cells under oxidative stress conditions
Source: FEBS Open Bio. 2018 Jan 2;8(2):219–33. doi: 10.1002/2211-5463.12360 (PMC5794457; doi:10.1002/2211-5463.12360)
Supplement: Supplementary file 4 — Table S1. miRNAs precursors and precursors variants coming from RNA‐Seq analysis. [file FEB4-8-219-s004.docx]

**Table S1.** miRNAs precursors and precursors variants coming from RNA-Seq analysis.

Data analysis highlighted that, throughout the whole experiment, 115 precursors, with or without variants, were detected between treated and untreated samples.

**Table S2.** mirPath KEGG and GO analysis.

**Table S3.** ClueGO detailed pathway analysis of miRNAs target genes from miRTarBase and microT databases.
